# Comparison of Wearable Trackers’ Ability to Estimate Sleep

**DOI:** 10.3390/ijerph15061265

**Published:** 2018-06-15

**Authors:** Jung-Min Lee, Wonwoo Byun, Alyssa Keill, Danae Dinkel, Yaewon Seo

**Affiliations:** 1 College of Physical Education, Kyung Hee University, Yougin 449-701, Korea; 2 College of Health, Kinesiology, and Recreation, University of Utah, Salt Lake, UT 84112, USA; won.byun@utah.edu; 3 School of Health and Kinesiology, University of Nebraska at Omaha, Omaha, NE 68182, USA; akkeill2@gmail.com (A.K.); dmdinkel@unomaha.edu (D.D.); 4 College of Nursing and Health Innovation, University of Texas at Arlington, Arlington, TX 76019, USA; Yaewon.seo@uta.edu

**Keywords:** wearable trackers, sleep monitors, sleep tracker

## Abstract

Tracking physical activity and sleep patterns using wearable trackers has become a current trend. However, little information exists about the comparability of wearable trackers measuring sleep. This study examined the comparability of wearable trackers for estimating sleep measurement with a sleep diary (SD) for three full nights. A convenience sample of 78 adults were recruited in this research with a mean age of 27.6 ± 11.0 years. Comparisons between wearable trackers and sleep outcomes were analyzed using the mean absolute percentage errors, Pearson correlations, Bland–Altman Plots, and equivalent testing. Trackers that showed the greatest equivalence with the SD for total sleep time were the Jawbone UP3 and Fitbit Charge Heart Rate (effect size = 0.09 and 0.23, respectively). The greatest equivalence with the SD for time in bed was seen with the SenseWear Armband, Garmin Vivosmart, and Jawbone UP3 (effect size = 0.09, 0.16, and 0.07, respectively). Some of the wearable trackers resulted in closer approximations to self-reported sleep outcomes than a previously sleep research-grade device, these trackers offer a lower-cost alternative to tracking sleep in healthy populations.

## 1. Introduction

The influence of sleep on individuals’ overall health is an important aspect of preventative care for many chronic diseases [1]. Previous studies have shown that physical fatigue, weakened immune systems, poor appetite regulation, lower mental alertness/memory function, and long-term health conditions (i.e., sleep apnea, heightened risk for stroke and coronary heart disease, type 2 diabetes) can be caused by poor sleep [1,2,3,4,5]. In particular, people who are obese and/or overweight have been associated with unfavorable sleep characteristics such as irregular sleep habits, short sleep duration, and low quality of sleep [6,7,8,9,10,11].

The ability to identify sleep patterns in individuals is important because they vary from population to population and are tied to many different health outcomes. Thus it is imperative to ensure measures used to identify sleep patterns are valid and reliable. Sleep research measurements vary depending on the feasibility of the methods. Subjective sleep monitoring is typically done with a sleep log or diary that is kept over any number of nights, depending on what is being measured. Interviews and questionnaires are other examples of subjective measurements used to obtain retrospective information on sleep behaviors. 

The “gold standard” for objective sleep monitoring is called polysomnography (PSG). This method typically takes place in a sleep lab. The participant being monitored wears various electrodes on their head and while they sleep their brain waves are detected and recorded. Extensive research on brainwaves during sleep has been conducted and it is understood that sleep contains specific stages, characterized by specific types of brain waves [12]. As PSG has high cost in resources, other methods of objective measurement are needed. The potential answer is the wearable tracker. These trackers are often worn on the hip or the wrist and use accelerometers and other sensors to gain information on the wearer’s movement. Older versions did not include the algorithms now available to detect sleep, and were typically removed during the night. New versions are equipped for detecting sleep, which opens up a whole new realm of objective sleep monitoring.

Several studies in research settings have reported on the validity of the wearable tracker on sleep measurement compared to PSG [13,14,15,16]. Zabotti et al. (2015) reported the total sleep time (TST) and wake after sleep onset (WASO) measured by the Jawbone UP showed good agreement with those measured by the PSG. They demonstrated 85.6%, 89.2%, and 73.8% of the sample were in the priori established satisfactory range for TST, WASO, and sleep efficacy (SE), respectively [17]. Cook et al. (2017) tried to quantify sleep with Jawbone UP3 and compared it to PSG, reporting inaccuracy of the Jawbone UP3 in sleep duration (mean difference: 20.5 min, *p* = 0.03) and efficiency (mean difference: 3.7%, *p* = 0.03) [13]. Cellini et al. (2013) studied the accuracy of two research-grade devices (i.e., actigraphy devices, AW-64 and GT3X, for sleep measurement with PSG, and reported the GT3X (kappa: 0.52) is a more valid and reliable device than the AW-64 (kappa: 0.28–0.46) [14]. 

However, most of this research has been conducted in a research setting and little is known about the quantitative comparison of the consumer wearable trackers and research-grade monitors (i.e., ActiGraph) in a free-living setting for measuring sleep. Therefore, the comparison of sleep measurement between the wearable trackers, including a research-grade monitor, and sleep diary as a purposeful criterion measure in a free-living condition was examined in the present study. 

## 2. Materials and Methods

### 2.1. Participants and Instrument

Participants were 19 years or older, had regular sleep patterns, and recruited from a Midwestern University. We defined “regular sleep patterns” as the participant’s ability to maintain a sleep schedule without the aid of medication screened by consensus sleep diary [15]. Individuals with a diagnosis of insomnia were excluded, as this sleep disorder could influence results obtained from an otherwise healthy population. Individuals who were pregnant were also excluded. This study was approved by the University of Nebraska Medical Center Institutional Review Board (IRB #080-15-EP). Seventy-nine healthy subjects signed the informed consent form to participate in this study. Participants were divided into two groups, detailed later, and descriptive characteristics by group and gender are shown in Table 1. The study population was 71% Caucasian, 22% Asian/Pacific Islander, 6% Hispanic/Latino, and 1% African American. 

### 2.2. Instrument

*ActiGraph GT9X Link (ActiGraph, Pensacola, FL, USA)*. The ActiGraph GT9X Link (35 mm × 35 mm × 100 mm, and a weight of 14 g) is a research-grade triaxial accelerometer. Depending on the version, the monitor can be worn at the waist, on the wrist, or the ankle. The monitor has been validated for sleep with adult and adolescent populations against laboratory PSG [16,18,19]. The raw data was downloaded using the ActiLife software (version 6.5.3, ActiGraph, Pensacola, FL, USA) and converted into Excel files for use with sleep analysis. The Sadeh and Cole-Kripke sleep algorithm was applied to analyze the data with the Tudor-Locke “default” for sleep period detection.

*SenseWear Mini armband (Jawbone, San Francisco, CA, USA)*. The SenseWear Mini Armband (62 mm × 55 mm × 13 mm, and a weight of 45.4 g) is a wearable tracker worn over the triceps of the wearer’s non-dominant arm. The monitor uses a triaxial accelerometer to capture movement, galvanic skin response (a change in the electrical resistance of the skin, i.e., sweating), skin temperature, and the rate at which heat is dissipated from the body. This monitor has been validated for sleep in adult populations with laboratory PSG [20]. Raw data were downloaded using the SenseWear software (version 8.1, Jawbone, San Francisco, CA, USA). Data from the variables pertaining to “Lying Down” and “Sleep” as well as the respective date/time stamps were exported to an Excel spreadsheet and separated into columns to represent the three nights. Unless a different sleep pattern was indicated in the participant’s sleep diary, the data were cut at noon to separate each night. The software uses minute-by-minute epochs for data analysis, and codes “Sleep” and “Lying Down” by using a “1” to indicate the presence of the action and a “0” to indicate the absence. 

*Basis Peak (Intel^®^, Santa Clara, CA, USA)*. The Basis Peak (36 mm × 273 mm × 27 mm, and a weight of 24 g) is a wrist-worn activity monitor, and has waterproof function up to 5ATM (atmospheres, a rating for water resistance), a triaxial accelerometer, skin temperature, galvanic skin response, and continuous heart rate using optical heart rate sensors (LED lights and an electro-optical cell). The Basis Peak also syncs wirelessly with the application (version 1.20.1, Intel, Santa Clara, CA, USA) on a mobile device to provide the user with information on their daily activity with activity trends over time. Because sleep duration and awakenings during the night were manually entered to Excel for each participant, screen shots from the online website under the user’s account, containing the participant’s sleep data over the study period were saved to that participant’s data file as a back-up.

*Fitbit Charge HR (Fitbit Inc., San Francisco, CA, USA)*. The Fitbit Charge HR (137 mm–57.5 mm × 21 mm wide and a weight of 25.8 g) is a wrist-worn activity monitor that measures user’s movement using a triaxial accelerometer, stairs climbed using an altimeter, and continuous heart rate using optical heart rate sensors. The monitor uses these measures to give the user information regarding step count, intensity, distance travelled, stairs climbed (increasing 10ft based on atmospheric pressure), calories burned, and TST. The band can also be wirelessly connected to the application (version 2.15.1, Fitbit, San Franscisco, CA, USA) on a mobile device or synced to a computer to track activity and sedentary patterns over time. To download data, the Fitbit was synced to its respective device and account. From the online website under the user’s account, screen shots were taken of any sleep data accrued over the study period, and the values on sleep data were manually entered to an Excel spreadsheet. 

*Jawbone UP3 (Jawbone, San Francisco, CA, USA)*. The Jawbone UP3 (140 mm–200 mm and a weight of 20 g) is a wrist-worn activity monitor that measures movement using a tri-axial accelerometer and resting heart rate using bioelectrical impedance sensors. The monitor uses this information to provide feedback on step count, intensity, and resting heart rate. The monitor does not have a screen on the band but can be wirelessly synced to the application (version 1.2.14, Jawbone, San Francisco, CA, USA) on a mobile device to view data and track trends over time. To download data, the monitor was synced to its respective device and account. Screen shots from the mobile application containing the participant’s sleep data over the study period were saved to that participant’s data file for manual entry into the Excel spreadsheet.

*Garmin Vivosmart (Garmin International Inc., Olathe, KS, USA)*. The Garmin Vivosmart (140 mm–200 mm and a weight of 19 g) is a wrist-worn activity monitor that measures movement using a tri-axial accelerometer. Step counts, distance travelled, sleep time, and energy expenditure are measured by the Garmin Vivosmart. The Garmin Vivosmart has a screen where the user can track activity in real-time or the monitor can be synchronized with a computer or the application (version 3.90, Garmin International Inc., Olathe, KS, USA) on a mobile device to track activity over time. Screen shots from the mobile application containing the participant’s sleep data over the study period were saved to that participant’s data file for manual entry into an Excel spreadsheet.

*Consensus Sleep Diary—Expanded.* This sleep diary was created through a collaborative effort by insomnia researchers, a project stemming from the 2005 Insomnia Assessment Conference, to address the lack of consistency in previously used sleep diaries [15]. Validity and reliability are rarely reported with sleep diaries due to the nature of the sleep assessment. However, this sleep diary has been standardized using focus groups with good sleepers, individuals with insomnia, and individuals with sleep apnea [15]. The diary features two sections, one to be filled out at night before going to bed and the other to be filled out in the morning. The participants were advised to complete the “morning” and “night” side immediately upon waking and before bed to reduce recall error. For data analysis, all sleep duration times were expressed in minutes.

### 2.3. Procedure

The length of participation was four days to obtain three full nights of data. All three nights of data collection were completed in the participant’s home to facilitate a “free-living” condition. At the first visit to the lab, the participants were informed of the benefits and risks associated with the study before signing the informed consent form. Once subjects completed the consent form and demographic questionnaire they were randomly allocated to Group 1 or Group 2. Group 1 participants wore the ActiGraph GT9X Link, SenseWear Mini Armband, Basis Peak, and Fitbit Charge HR. Group 2 participants wore the ActiGraph GT9X Link, the Garmin Vivosmart, and the Jawbone UP3. Date of birth, and handedness were collected from the demographic questionnaire, and used to initialize and synchronize each monitor with participants’ weight and height measured. The monitors that were worn on the wrist were counterbalanced (i.e., worn in a different order) from subject to subject. Because the SenseWear Mini Armband was worn on the upper arm, counterbalancing did also apply to this monitor on the wrist. Each participant wore their assigned monitors over their participation period, except when showering or swimming, and completed the sleep diary each day. Subjects were instructed on how to care for their monitor during the study period. Additionally, subjects were provided a sleep diary and instructed on how to properly complete their sleep diary. After three nights, the participant returned the monitors and their sleep diary to the lead researcher. 

### 2.4. Data Analysis

Variables of interest for statistical analysis were TST, time spent in bed (TIB), SE, and WASO. Sleep onset latency (SOL) was excluded for analysis since the wearable trackers used in the present study do not provide SOL assessment. Except for the SE, which is expressed as a percentage ([TST/TIB] × 100), all other variables were calculated in minutes, as opposed to hours to increase the accuracy of the values. TST was defined as the duration of sleep between the onset of sleep and the final awakening. TIB was defined as the duration of time between “getting into bed” and “getting out of bed for the day.” WASO was defined as the time spent awake during a night of sleep. 

If a monitor detected several separate sleep periods in one night, the information was combined to create a single night’s sleep. If a band needed sleep “verified” by the app user, or did not detect sleep but allowed the app user to add their sleep, the respective night in the participant’s sleep diary was input into the application by the researcher, as this mimics the way a user would interact with their own app. For the analysis of WASO for all bands, zeros (indicating the participant did not wake during the night) were re-coded to 0.1 to avoid dividing by zero when performing the mean absolute percentage error (MAPE) calculations.

Descriptive statistics were performed to summarize the participants’ demographic information as well as sleep variables. Pearson correlation coefficients for each variable were calculated for the total study population from each monitor and then compared to the sleep diary. Glass’ delta was calculated to express the effect size, which was included in the total study population to determine statistical significance [21]. The MAPE was calculated to quantify the absolute difference between each monitor and the sleep diary values. Bland–Altman plots were created to visually inspect bias in the data. Equivalence testing was used to determine whether the mean values of the sleep measurement fell within the 10% equivalence zone from the reference sleep diary. All of the statistical analyses were performed with SAS 9.4 (SAS Institute Inc., Cary, NC, USA)

## 3. Results

In total, 79 subjects were recruited to participate in this study. The age range of the participants was 19–66 years with a mean age of 27.6 ± 11.0 years. Participant BMI ranged from 19.4–39.7 kg·m^−2^ with a mean of 25.3 ± 4.6 kg·m^−2^ (Table 1). 

One subject was excluded from the data analysis because the participant did not fill out the sleep diary. Of the remaining 78 participants, 18 subjects had one night of data and 60 had three nights of data. Mean values for TST, TIB, SE (%), and WASO are shown in Table 2.

Table 3 shows the correlations between the sleep diary and each activity monitor, separated by sleep variable, and the corresponding effect size (Glass’ delta). Because analyzing the difference of sleep measurement between the monitor and sleep diary was the main goal of the present study, a lower value of effect size indicates the better validity of sleep measurement. Therefore, it is important to note that while some pairs suggest a moderate correlation, the large effect size would suggest that the strength of the relationship may not be as significant. The smallest value of effect size was 0.07 (sleep diary TIB vs. Jawbone UP3 TIB), and the greatest value of effect size was 1.21 (sleep diary TST and TIB vs. ActiGraph GT9X Link with Sadeh algorithm TST and TIB).

**Figure 1:**
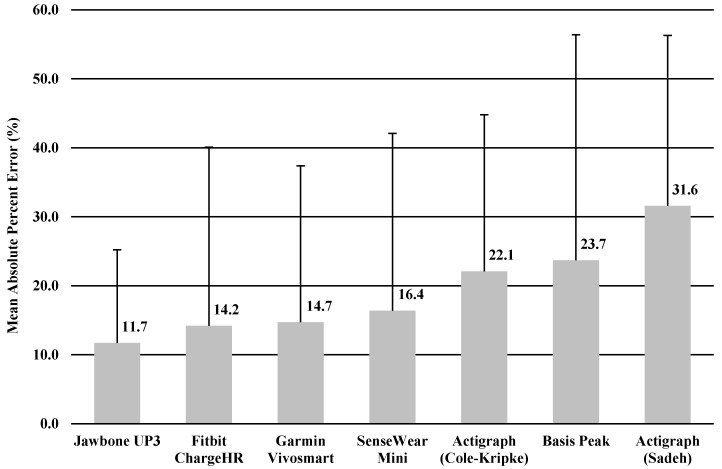
Mean absolute percentage error: total sleep time.

**Figure 2:**
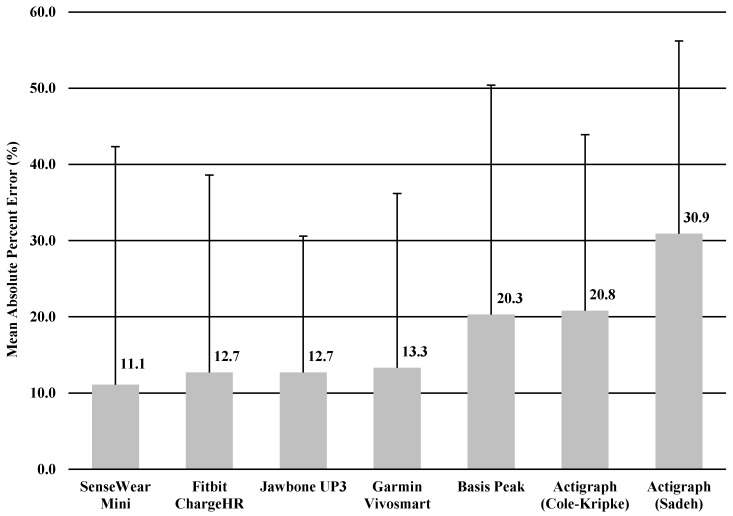
Mean absolute percentage error: time in bed.

The MAPE was calculated for TST and TIB based on the result of significant correlation of sleep variables between the monitor and sleep diary. MAPEs for TST and TIB were shown in Figure 1 and Figure 2, ranging from 11.7% to 31.6% for TST, and 11.1% to 30.9% for TIB. The Jawbone UP3 showed the lowest value in TST (11.7%), and the SenseWear Mini Armband showed the lowest value in TIB (11.1%). 

**Figure 3:**
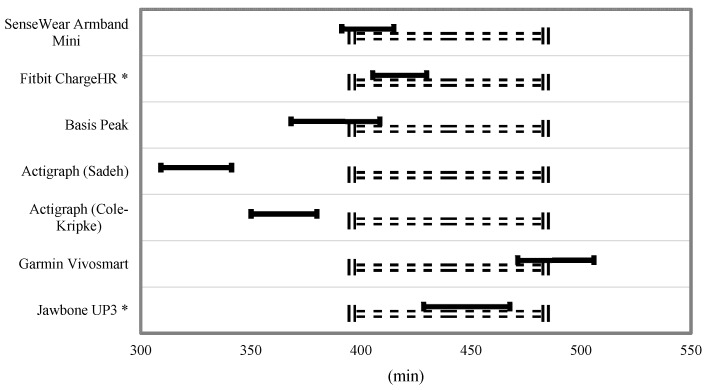
Equivalence testing for total sleep time. * Within the 10% equivalence zone. Dashed lines indicate proposed equivalence zone (±10% of the mean). Dark bars indicated the 90% confidence for a mean of the estimated total sleep time.

**Figure 4:**
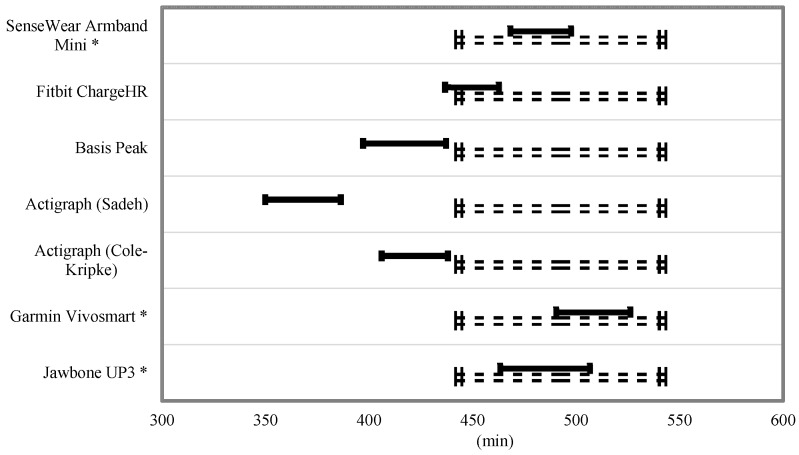
Equivalence testing for time in bed. * Within the 10% equivalence zone. Dashed lines indicate proposed equivalence zone (±10% of the mean). Dark bars indicated the 90% confidence for a mean of the estimated time in bed.

Equivalence testing for TST and TIB is shown in Figure 3 and Figure 4. For the TST, the Fitbit Charge HR and Jawbone UP3 were the only monitors to fully fall within the 10% equivalence zone. The SenseWear Mini Armband, Basis Peak, and Garmin Vivosmart overlapped this zone by a small margin. For the variable TIB, the SenseWear Mini Armband, Garmin Vivosmart, and Jawbone UP3 bands fell within the 10% equivalence zone. 

**Figure 5:**
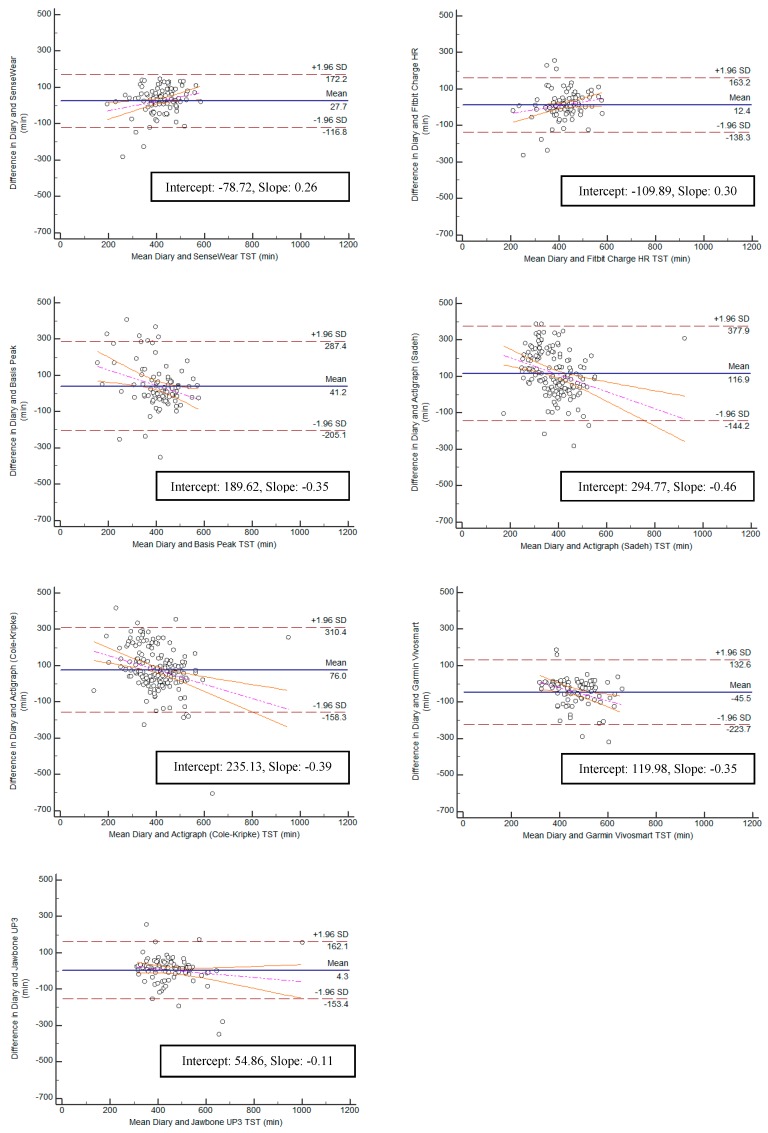
Bland–Altman plots for all monitors: total sleep time.

**Figure 6:**
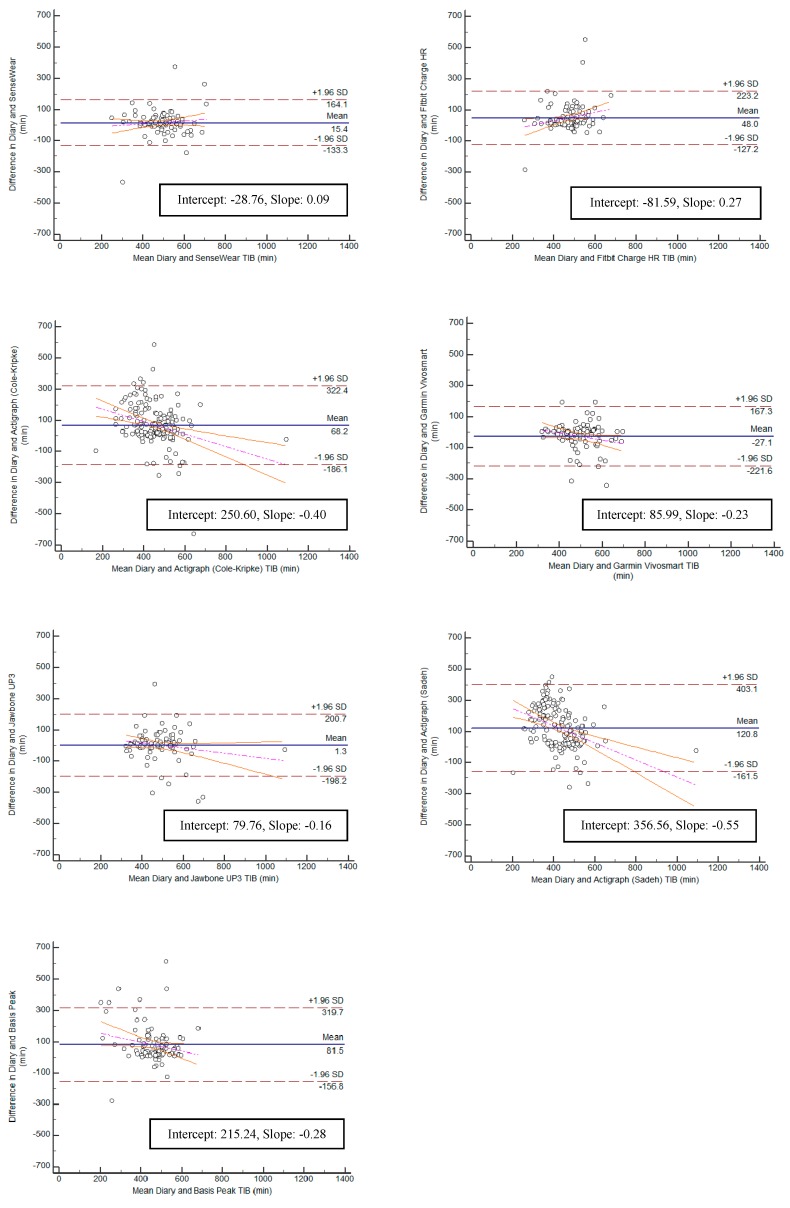
Bland-Altman plots for all monitors: Time in Bed.

Bland–Altman plots were created for the variables TST and TIB, and can be found in Figure 5 and Figure 6. Bias within the sample for TST ranged from the lowest having a slope of 0.11 (Jawbone UP3; y-intercept = 54.86) to the highest with a slope of −0.46 (ActiGraph GT9X Link-Sadeh; y-intercept = 294.77). Bias within the sample for TIB ranged from the lowest having a slope of 0.09 (SenseWear; y-intercept = −28.76) to the highest with a slope of −0.55 (ActiGraph GT9X Link-Sadeh; y-intercept 356.56) SE and WASO were eliminated from the equivalence testing based on poor Pearson correlation values.

## 4. Discussion

In the current study, we performed a descriptive analysis of the sleep measure of widely used accelerometer-based wearable trackers and a research-grade device for measuring sleep behaviors in healthy young adults in a free-living environment. We conducted 24-h monitoring for 3 consecutive nights in order to test whether six wearable trackers were able to assess sleep as compared with a sleep diary. We purposely used a sleep diary as a criterion measure in order to make comparison easier. Overall, the comparison was relatively similar among the four trackers, with the Jawbone UP3, Fitbit Charge HR, Garmin Vivosmart, and Sensewear Mini providing somewhat higher comparability than the ActiGraph (Cole–Kripke), ActiGraph (Sadeh), and Basis Peak when examining TST and TIB. Further, we observed comparable values between the research-grade device (i.e., Actigraph) and the wearable trackers in estimating TST and TIB. Measures of TST and TIB varied across the board but mostly ranged from minimal errors of MAPE (11.1%) to maximal errors of MAPE (31.6%) with the sleep diary. 

The results of this study appear consistent with other studies regarding actigraphy and sleep measures. In previous studies that have compared actigraphy to PSG, results consistently show that wearable trackers have a high sensitivity (the tracker’s ability to correctly identify sleep) and low specificity (the tracker’s ability to correctly identify wake) [16,22,23]. While we were unable to assess sensitivity and specificity in our study (as it requires the ability to compare epoch-to-epoch between methods), we were able to see results that suggest a similar association. We found, depending on the tracker, low to moderate significant correlations between the diary and the tracker for TST and TIB, similar to a study by Arora et al. (2013) and Evans et al. (2011). Our findings are also consistent with those studies citing activity trackers’ poor specificity due to the poor correlations seen between the sleep diary and the trackers for WASO [24,25]. 

In two separate studies lead by de Zambotti and colleagues [22,26], the Jawbone Up and Fitbit Charge HR tracker were validated against PSG. In the 2015 study with the Jawbone UP, the researchers found high sensitivity in sleep detection (0.97), which mirrors our Jawbone UP3 results for TST as the Jawbone UP3 had the smallest effect size, the lowest error, and the least bias. In the 2016 study, the researchers found the Fitbit Charge HR reported TST similarity within 30 min of the reported TST from PSG. Sensitivity with the Fitbit was reported to be 0.97 and specificity to be 0.42. Similarly to these studies, our study also found greater discrepancies between reported variables during nights that appeared to consist of more disrupted sleep (i.e., the sleep diary reported 7 h of sleep and each band would report between 2 and 7 h of sleep for that night).

Results from our study regarding the Basis Peak do not reflect previous research using the Basis activity tracker. Results reported from Basis Science (San Francisco, CA, USA), compared their Basis B1 Band—the activity tracker preceding the Basis Peak—to PSG. The researchers reported in their preliminary results that the advanced sleep analysis algorithm demonstrated “excellent agreement with polysomnography data for sleep duration (4.3% mean difference)” [27]. Because they had compared the Basis with PSG, we expected to see a strong association between sleep time and diary reported sleep time. However, when compared with the sleep diary in our study, the Basis Peak showed poor agreement with reported TST (23.7% mean difference). This difference in results could stem from the fact that there were more missed nights of sleep recording for the Basis Peak than the other consumer bands. The apps used by the Fitbit Charge HR, Jawbone UP3, and Garmin Vivosmart allow users to interact with the app by confirming sleep times. The Basis Peak app does not allow users to do this, so if movement or some other factor during the night did not allow the band to detect sleep when the algorithm was applied, that night contained no sleep data. 

Shin, Swan, and Chow (2014) assessed the validity of the SenseWear against PSG at different ambient temperatures. Their main finding was that the SenseWear was valid at 17 °C and 22 °C for WASO, SE, and TST, but significantly underestimated TST and overestimated WASO at 29 °C [20]. We did not assess ambient room temperature during sleep in our study, but we did find that the SenseWear was moderately correlated with the sleep diary for TST and had a poor correlation with WASO. Interestingly for our study, the SenseWear showed stronger correlation and equivalence with TIB (lying down). 

Overall, we found that the SenseWear, Fitbit Charge HR, Jawbone UP3, and Garmin Vivosmart can be valid measures of TST and TIB when compared with a sleep diary in a healthy adult population in a free-living setting. However, these trackers cannot be considered valid regarding wake times during a night of sleep. In this population, the Cole–Kripke algorithm for the Actigraph performed better than the Sadeh algorithm; however, neither algorithm was shown to be the most strongly correlated or equivalent to the sleep diary than the other trackers, and both showed large effect sizes. The potential success of the Cole–Kripke algorithm could be due to the older age of the population with which the algorithm was created, as opposed to the Sadeh algorithm, which included children and adolescents, as well as adults. 

This research provides insight into the comparability of the sleep measure of different wearable trackers. The increasing popularity of these trackers among the general public, wellness programs, and even clinical settings makes this information imperative, as the users are trusting these devices to provide them with information about their daily sleep patterns, potentially in the hopes to make positive lifestyle changes. In addition, because some of the wearable trackers (Jawbone UP3, Fitbit Charge HR, and Garmin Vivosmart) resulted in closer approximations to self-reported sleep duration than a previously sleep-validated research monitor (ActiGraph), these monitors offer a lower-cost alternative to tracking sleep in healthy populations.

The present study supports the incorporation of some wearable trackers into research applications by inclusion of a reasonable sample size and examination of a variety of wearable trackers that are currently available in the market. Another strength was the inclusion of a research-grade monitor which enabled for a more direct comparisons with the wearable trackers. The results of the study add to the existing literature on sleep monitoring and is one of the first to examine the comparability of these wearable trackers in free-living conditions. In addition, this study is the first study to utilize the novel statistical method (i.e., equivalence testing) typically utilized in validation research on wearable trackers measuring sleep variables. As equivalence testing is intended to conclude how closely one measure equates to another measure, it is a more appropriate analytic approach for this type of validation research. However, this study has some limitations. First, the measures used bring their own short-comings and trackers were not compared with the PSG (i.e., gold-standard measure). Wearable trackers do not measure sleep directly, sleep algorithms for Actigraphy are created based on (well-researched) assumptions about physical movement, sedentary activity, and sleep behavior. Because of this, participants who toss and turn frequently during sleep skew the data due to poor sleep detection and several participants had missing nights of data from some of the bands (i.e., ActiGraph and Basis Peak). Second, by using a sleep diary, we are assuming that the participants followed the protocol described to them at the first lab visit and filled out the diary immediately after waking and reported their sleep activity as accurately as possible. In addition, these wearable device manufacturers can change their algorithm at any time without consumers’ and researchers’ notification. Participants in this study consisted of primarily healthy, White, college-age individuals. As such, these findings may not be generalizable to older adults and ethnic minorities. 

In the future, more research is needed to compare these wearable trackers against the gold standard (PSG) in a laboratory and free-living conditions. Due to the popularity of these wearable trackers, research is also warranted in special populations, such as: adolescents, elderly, and sleep disordered populations.

## 5. Conclusions

Some of the wearable trackers (Jawbone UP3, Fitbit Charge Heart Rate, and Garmin Vivosmart) resulted in closer approximations to self-reported sleep outcomes than a previously sleep research-grade device, these trackers offer a lower-cost alternative to tracking sleep in healthy populations. As technological advances in wearables will constantly offer more feasible and reliable alternatives for measuring sleep patterns, researchers and practitioners need to be informed on the comparability of these wearable trackers that have significant potential for research and practical applications for measuring sleep.

## Figures and Tables

**Table 1:** Descriptive characteristics of participants (*n* = 78).

		Group 1		Group 2		Total
		Male (*n* = 19)	Female (*n* = 19)	Male (*n* = 17)	Female (*n* = 23)	
Age (Year)	Mean ± SD	30.1 ± 14.2	27.1 ± 11.3	27.9 ± 7.2	26.3 ± 10.1	27.6 ± 11.0
	Range	19–66	20–65	22–47	19–65	19–66
Height (cm)	Mean ± SD	17.9 ± 6.3	166.2 ± 7.0	179.8 ± 6.8	162.7 ± 6.2	171.3 ± 10.2
	Range	170.2–194.3	154.9–182.9	167.6–195.6	152.5–180.3	152.5–195.6
Weight (kg)	Mean ± SD	87.0 ± 18.9	68.7 ± 12.2	84.4 ± 28.8	71.0 ± 18.1	77.3 ± 21.0
	Range	64.9–129.5	49.0–92.3	64.9–192.5	51.6–130.0	49.0–192.5
BMI (kg∙m^−2^)	Mean ± SD	26.8 ± 5.5	24.8 ± 4.2	23.4 ± 2.4	25.7 ± 4.8	25.3 ± 4.6
	Range	20.5–39.7	19.4–34.8	20.3–28.2	21.3–38.8	19.4–39.7

BMI = Body Mass Index.

**Table 2:** Mean values of sleep variables (min).

	N*	TST	TIB	SE (%)	WASO
		Mean ± SD	Mean ± SD	Mean ± SD	Mean ± SD
Diary	195	439.8 ± 94.6	492.4 ± 101.9	88.6 ± 10.8	20.9 ± 33.0
SenseWear Armband Mini	99	403.1 ± 70.6	482.8 ± 87.7	84.1 ± 8.5	79.0 ± 49.3
Fitbit Charge HR	98	417.6 ± 73.1	449.7 ± 77.4	93.3 ± 3.2	31.4 ± 16.8
Basis Peak	93	388.4 ± 116.9	417.2 ± 116.3	92.2 ± 18.8	N/A**
ActiGraph (Sadeh)	163	325.2 ± 124.0	368.1 ± 140.9	88.3 ± 5.9	42.0 ± 25.8
ActiGraph (Cole-Kripke)	183	365.1 ± 122.2	422.1 ± 130.8	87.7 ± 5.8	50.6 ± 27.1
Garmin Vivosmart	96	488.6 ± 102.3	508.2 ± 105.6	96.4 ± 5.6	19.7 ± 32.2
Jawbone UP3	92	448.1 ± 113.1	484.9 ± 125.1	92.5 ± 5.2	36.9 ± 30.9

TST = Total Sleep Time; TIB = Time in Bed; SE = Sleep Efficiency; WASO = Wake after Sleep Onset; N* = nights of data; N/A** = Basis Peak is not capable of assessing WASO.

**Table 3:** Correlation matrix and effect sizes.

		SenseWear Armband Mini	Fitbit Charge HR	Basis Peak	ActiGraph (Sadeh)	ActiGraph (Cole-Kripke)	Garmin Vivosmart	Jawbone UP3
Sleep Diary	TST	0.57 **	0.55 **	0.28 **	0.27 **	0.41 **	0.52 **	0.73 **
	Effect Size	0.39	0.23	0.54	1.21	0.79	0.52	0.09
	TIB	0.66 **	0.48 **	0.36 **	0.32 **	0.39 **	0.49 **	0.64 **
	Effect Size	0.09	0.42	0.74	1.22	0.69	0.16	0.07
	SE	−0.09	−0.03	−0.09	−0.06	−0.04	0.18	0.26 *
	Effect Size	0.41	0.44	0.33	0.03	0.08	0.72	0.36
	WASO	0.01	0.09	N/A	0.08	−0.02	0.05	0
	Effect Size	1.76	0.32	N/A	0.64	0.90	0.04	0.48

TST = Total Sleep Time; TIB = Time in Bed; SE = Sleep Efficiency; WASO = Wake after Sleep Onset; ** Correlation is significant at the 0.01 level (2-tailed); * Correlation is significant at the 0.05 level (2-tailed).
